# Recent Advances in Understanding the Sex Pheromone‐Mediated Communication of Diatoms

**DOI:** 10.1002/cplu.202500224

**Published:** 2025-06-30

**Authors:** Franziska Alessandra Klapper, Georg Pohnert

**Affiliations:** ^1^ Institute for Inorganic and Analytical Chemistry, Bioorganic Analytics Friedrich Schiller University Jena Lessingstrasse 8 Jena 1 07743 Germany; ^2^ Trasfer Group Antiinfectives Leibniz Institute for Natural Product Research and Infection Biology (HKI) Jena 07745 Germany; ^3^ Fellow Group Plankton Community Interaction Max Planck Institute for Chemical Ecology Hans‐Knöll‐Straße 8 Jena 07745 Germany

**Keywords:** biofilms, cyclic peptides, diatoms, microalgae, pheromones, sexual reproduction

## Abstract

Diatoms are prolific unicellular microalgae, contributing ≈20% of global photosynthetic CO_2_ fixation. These algae form the base of the marine food web. They are also widely distributed in freshwater and utilized in aquaculture. Despite their ecological and economic significance, many fundamental aspects of diatom biology, including their sexual reproduction, remain poorly understood. This review highlights recent advances in unraveling the chemical signaling essential for diatom sexual reproduction and introduces how natural products chemistry and marine microbiology synergize to unravel novel chemical communication strategies. Diatoms communicate through a sophisticated pheromone‐based “language” that synchronizes cell cycles, regulates the physiology, and guides cells to their mating partners. The structural elucidation of diatom pheromones becomes possible with emerging model organisms and the development of analytical approaches to detect these compounds at extremely low active concentrations. Breakthroughs in comparative metabolomics, combined with reliable bioassays, have enabled the identification of the first pheromones. However, only the adaptation of novel labeling techniques and sensitive NMR experiments allows the determination of the pheromone structures. With these structural insights and the availability of genetic resources, a new research field is emerging—spanning evolutionary studies to the potential manipulation of natural diatom populations and applications in aquaculture.

## Graphical abstract

Pheromones control the sexuality and mate finding in diatoms, a group of highly abundant unicellular marine algae. The diatom pheromones presented here are elucidated using a metabolomics‐based approach. This review introduces new structures, methods, and conceptual advances and discusses future perspectives in diatom research.

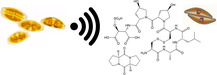

## 1 Introduction

Humans primarily communicate via sounds and sights. Another highly specific yet intricate form of communication, which plays a minor role in human interactions but is crucial for many other organisms, is chemical signaling. It represents the oldest form of communication between organisms, utilized throughout all domains of life from microbes to mammals.^[^ ^1^, ^2^ ^]^ In aquatic systems, which account for 90% of habitable space on earth, the involved chemical signals have the potential to shape whole community structures,^[^ ^3^ ^]^ which ultimately affects the marine food web. Making use of highly specific signal molecules and their bioactivities is of economic and social interest, since many species that are controlled by chemical mediators bear ecological and economic importance.

Living throughout the euphotic zone (the upper layer of a body of water, where sunlight is sufficient for photosynthesis) of the oceans and freshwater ecosystems, algae are vital not only for the marine ecosystem but also for the biogeochemical carbon cycle and cycling of nutrient elements, such as nitrogen and silicon.^[^ ^4^, ^5^ ^]^ This makes algae key players in maintaining the health and function of our planet, and providing core ecosystem services in the oceans. Comprising more than 100 000 species, diatoms constitute the most diverse group of algae,^[^ ^6^ ^]^ inhabiting marine as well as freshwater ecosystems and muddy sediments at coastal areas. Due to their wide distribution, they are responsible for 40% of the marine primary net production.^[^ ^7^ ^]^ Moreover, diatoms have a great impact on the sulfur and silica cycle,^[^ ^8^ ^]^ the latter resulting from their unique silica cell wall.

During their complex life cycle, these microalgae need to interact through chemical signals, which are the main topic of this contribution. These signals are unique in their chemistry and in their ability to synchronize marine microbial communities. It has yet to be explored if they are prototypic for marine diatoms or if a species‐specific pheromone family will emerge during further investigations. We review here, after a short overview on algal sex pheromones in general, the progress made in the last decade on diatom chemical communication and refer to earlier reviews for a comprehensive literature review through 2014.^[^ ^9^, ^10^ ^]^ We aim to introduce the fundamental concepts in chemistry and biology that are required to successfully merge the disciplines and to obtain insight into an intricate aquatic signaling chemistry.

## 2 Overview of Sex Pheromones in Algae

Most algae have complex life cycles in which vegetative forms, which are dominant in the environment, can produce gametes that fuse to form a zygote that can develop over multiple stages to the vegetative form again.^[^ ^9^, ^10^, ^11^ ^]^ During this process, pheromones are involved in synchronization and mate finding in different ways. It is striking that the involved pheromones are structurally highly diverse, even if they all serve the support of mate finding and synchronization of sexuality under water. While brown algae utilize highly unpolar fatty acid‐derived hydrocarbons that are likely enriched in hydrophobic receptors, green algae use glycosylated, more polar metabolites, and diatoms rely on peptides. In the following, we briefly introduce the pheromone function and chemistry of green and brown algae to contextualize the pheromone chemistry of diatoms. For a full overview on the sex pheromone chemistry of algae, refer to the review of Frenkel et al.^[^ ^9^ ^]^

**Figure 1:**
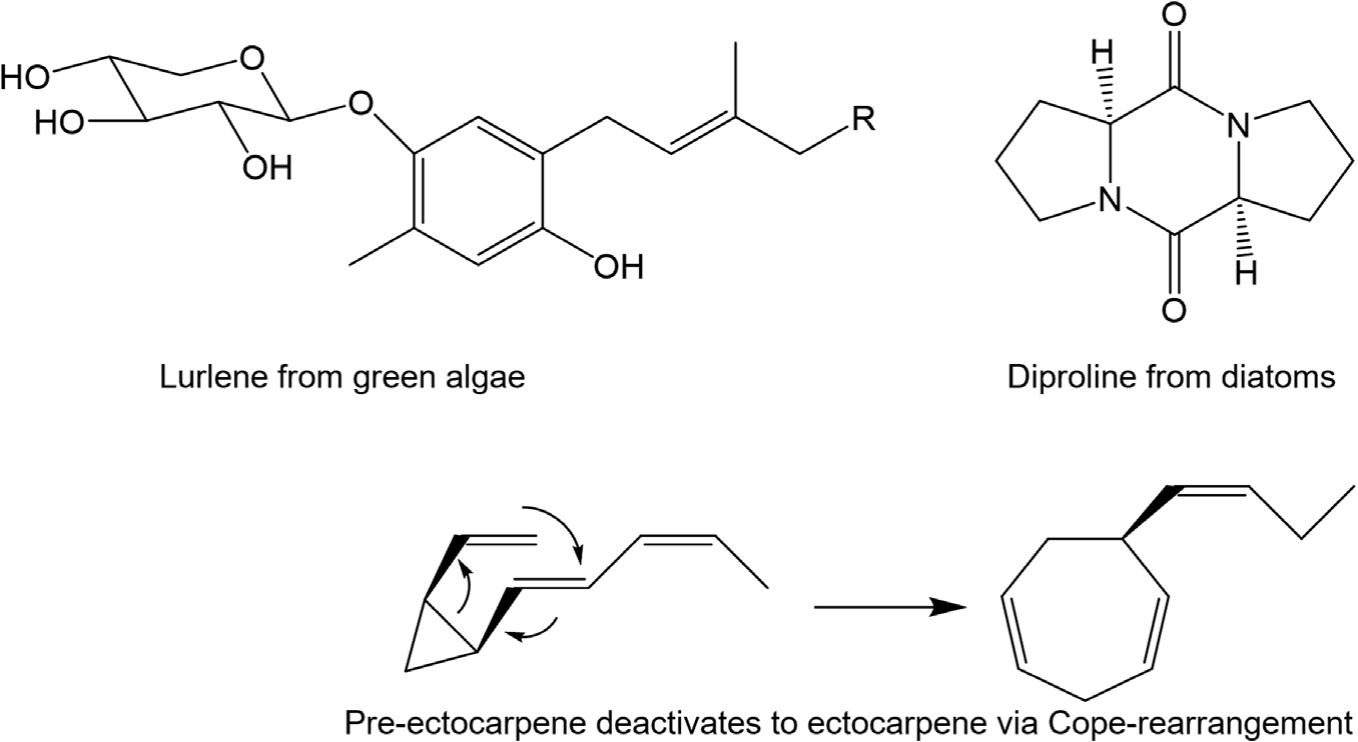
Selected algal pheromones from green and brown algae, as well as diatoms. Diversity in chemical nature and size is represented (R = C_11_H_18_COOH). Pheromones comprise complex glycoproteins as well as small volatile hydrocarbons. Adapted with permission.^[^ ^9^ ^]^ Copyright 2014, Wiley.

In brown and green algae, pheromones have been identified early, mainly based on gas chromatography/mass spectrometry (GC/MS)‐data and synthesis of postulated structures, but only much later, in diatoms, a first attraction pheromone was identified (**Figure** **1**).^[^ ^9^ ^]^

Primer pheromones induce a change in physiology, like the formation of gametes, whereas releaser pheromones trigger a behavioral change, like the attraction of motile partners. The first algal sex pheromone structure that was identified was ectocarpene, an unsaturated fatty acid‐derived C_11_‐hydrocarbon that triggers chemoattraction of male brown algal gametes to females in *Ectocarpus siliculosus*.^[^ ^12^ ^]^ However, more than 20 years later, Boland and co‐workers found the actual active compound to be the thermolabile preectocarpene that exhibits a substantially higher activity even at 10^9^‐fold dilution compared to ectocarpene.^[^ ^13^ ^]^ This pheromone is inactivated by a pericyclic cope reaction, a process that is controlled entirely by temperature. This inactivation guarantees that gametes are not misguided by old pheromone traces. Ectocarpene is also the attracting pheromone in the brown seaweed *Mutimo cylindricus*, where it converts gamete behavior from phototaxis to chemotaxis mediated by cAMP/Ca^2+^‐dependent signaling.^[^ ^14^ ^]^

In green algae, sexual reproduction is often triggered by environmental cues and accompanied by pheromones. Gametogenesis in *Chlamydomonas reinhardtii* is induced by nitrogen deficiency, followed by the production of the glycoprotein agglutinin to adhere gametes.^[^ ^15^ ^]^ In many *Volvox* species, heat shock causes the production of high‐molecular‐weight glycoproteins that elicit sexual reproduction and induce gametogenesis.^[^ ^16^ ^]^ In contrast to other algae, most encounters between green algal gametes are not guided by pheromones but happen by chance. The only identified attractant in green algae so far is lurlene, secreted by motile gametes of the green algae *Chlamydomonas allensworthii* to attract a compatible partner.^[^ ^17^ ^]^ The pheromone system of another green algal species, the unicellular alga *Closterium peracerosum‐strigosum‐littorale* complex, has been studied in detail.^[^ ^18^ ^]^ In this species, nitrogen depletion activates a reciprocal signaling cascade that involves at least two pheromones. A protoplast‐release‐inducing protein (PR‐IP) is exuded by mt^+^ cells and triggers the release of gametes from mt^−^ cells (note: in algae, the sexes are not defined as male or female but as mating types mt^+^ and mt^−^).^[^ ^19^ ^]^ The secretion of this pheromone is induced by a PR‐IP inducer pheromone constitutively secreted by mt^−^ cells. Both pheromones were suggested to be multifunctional by additionally triggering the secretion of mucilage.

## 3 Pheromones in the Life Cycles of Diatoms

**Figure 2:**
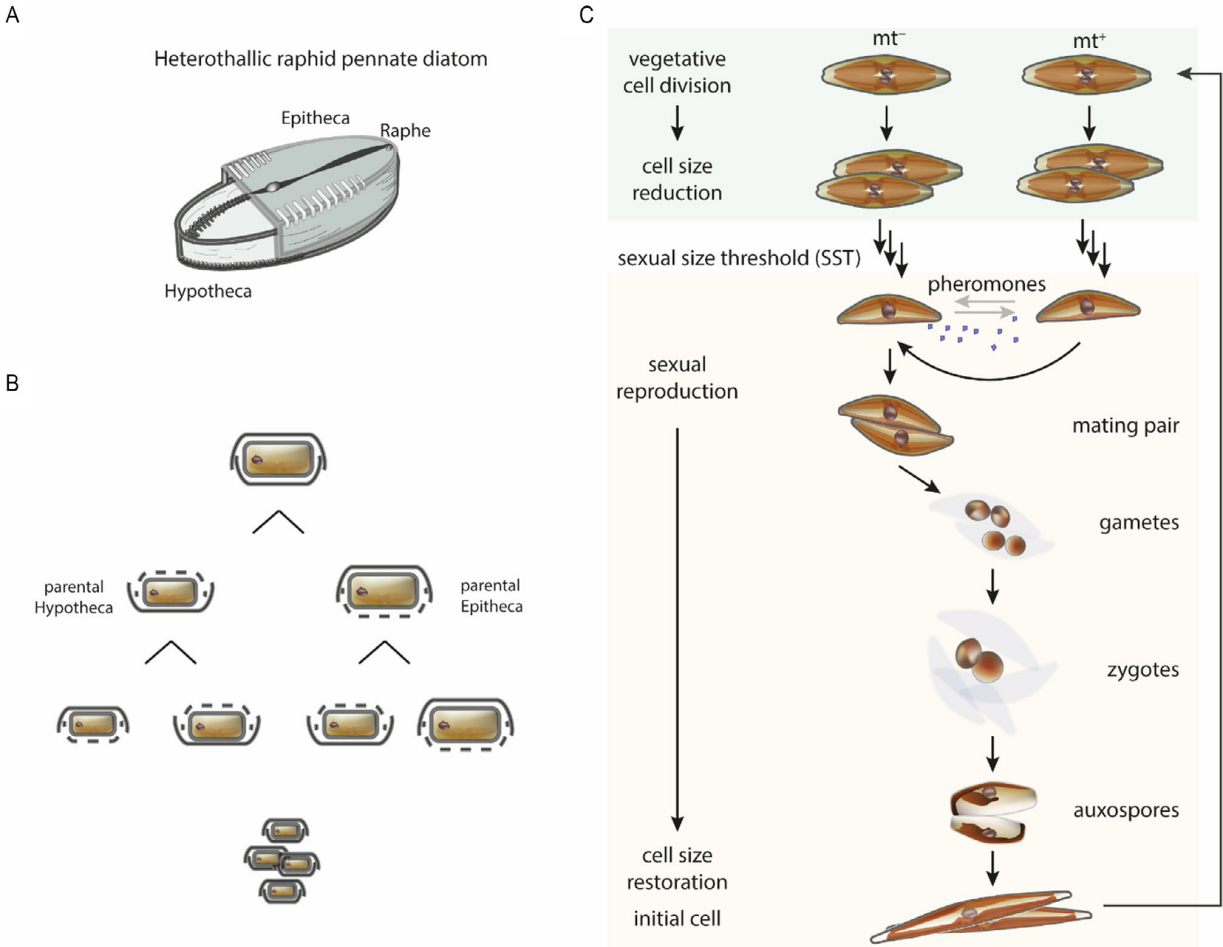
Life cycle of diatoms. A) Diatom cell walls are built out of silicate. This glass‐like structure is rigid and protects the cells. B) Most of these microalgae reproduce predominantly by mitotic cell division, building their new cell wall within the parent cell. This process leads to a gradual decrease in cell size. C) Upon reaching a species‐specific SST, diatoms can switch to sexual reproduction to restore their cell size. Life cycle strategies vary between species—we depict here the model diatom *S. robusta*. Upon reaching the SST, genetically different mating type (mt) cells can be distinguished by their behavior. Mt^+^ is defined as the searching partner whereas mt^−^ is attracting the other partner by means of pheromones. Pair formation (and thus sex) results in gamete formation and fusion into a zygote. This grows into auxospores and develops into a cell of an initial big size. Adapted with permission.^[^ ^31^ ^]^ Copyright 2022, Springer.

Despite their huge diversity in morphology, nearly all diatoms share a unique biomineralized cell wall, the frustule, which is formed upon precipitation of silicic acid.^[^ ^20^ ^]^ Vegetative growth of diatoms by cell division is quicker compared to other microalgae because less energy is required for the synthesis of a frustule than the synthesis of an organic cell wall, partly explaining the vast abundance of these microalgae.^[^ ^21^ ^]^ The amorphous silica cell wall of a diatom is constituted of two thecae that are slightly different in size but fit perfectly into each other (**Figure** **2**A). With every mitotic division, each daughter cell inherits one of the parental theca. In most species, the hypotheca is slightly smaller than the epitheca, thus, daughter cells formed from the parental hypotheca have a smaller cell size. This results in the characteristic decrease of average cell size of a population upon divisions known as the MacDonald–Pfitzer rule (Figure 2B).^[^ ^22^, ^23^, ^24^ ^]^ To escape death and restore the maximum cell size, cells switch to short episodes of sexual reproduction that can last from hours to several days.^[^ ^25^ ^]^ Over the last years, evidence accumulates that many of the processes that are involved in sexual reproduction are under the control of pheromones.^[^ ^26^, ^27^, ^28^, ^29^, ^30^, ^31^ ^]^

For over 20 years, *Seminavis robusta* has been established as the model organism to study sexual reproduction in diatoms. This diatom has a life cycle dominated by rounds of mitotic cell division that result in size reduction of the cells.^[^ ^32^ ^]^ Once the size threshold is reached, cells become sexually competent, and the two mating types can be distinguished by their characteristic behavior. While cells attracting the other partner are termed mating type^−^ (mt^−^), cells of mating type^+^ (mt^+^) are searching for a compatible partner and move toward it.^[^ ^33^, ^34^ ^]^ This unique mate finding behavior in pennate diatoms is facilitated by excretion of extracellular polymeric substances through an elongated cell wall slit, termed raphe. This enables cells to glide back and forth with quasiinstantaneous reversals, allowing a mate‐finding process that is synchronized and guided by pheromone gradients emitted by the mating partner.^[^ ^35^, ^36^, ^37^ ^]^ Once cells have met, they associate to form a mating pair, develop via gametes, zygotes, and auxospores to cells of the initial size (Figure 2C).

**Figure 3:**
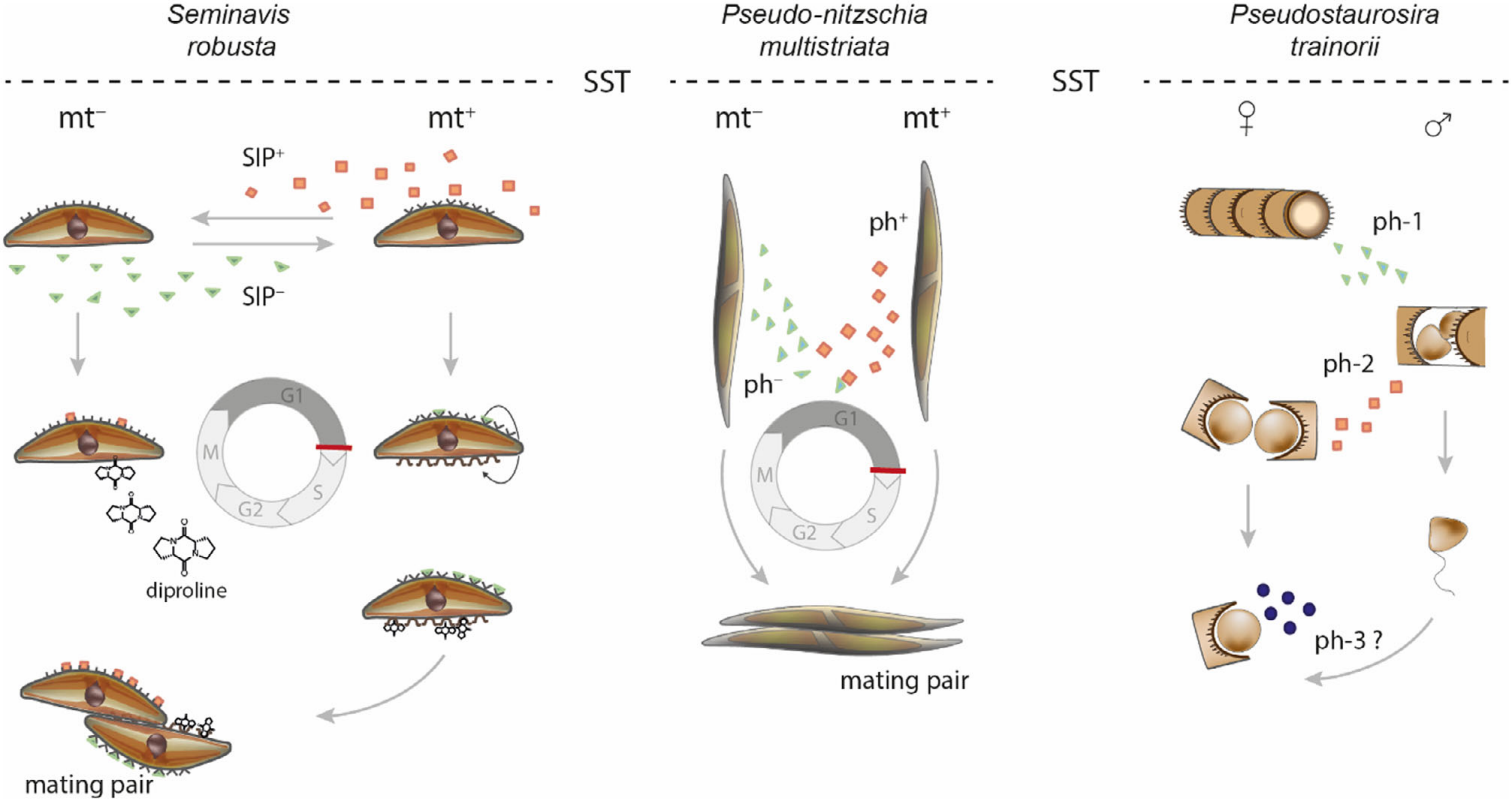
Pheromone systems of selected pennate diatoms. The diatom *S. robusta* has a reciprocal pheromone system with SIPs that synchronize the cell cycle of the mating partner. In addition, these SIPs induce diproline production and diproline receptor formation in the opposite mating types (left). Mating type^+^ (mt^+^)‐cells can sense diproline and glide toward a diproline‐producing partner (mt^−^) to pair. The middle panel shows that the planktonic diatom *Pseudo‐nitzschia multistriata* also uses hitherto unknown pheromones (ph^+^/ph^−^) to synchronize the cell cycle but pair formation is not chemically mediated. The araphid diatom *Pseudostaurosira trainorii* utilizes three uncharacterized pheromones (ph‐1/2/3) to step‐wise sexualize the mt^+^ and mt.^−^ cells (right). Adapted with permission.^[^ ^31^ ^]^ Copyright 2022, Springer.

Since sexual reproduction is a crucial process for the survival of the population, cells need powerful regulatory mechanisms to synchronize this event. This synchronization is guaranteed by chemical cues (pheromones) that regulate the timing of sexual reproduction and the mate‐finding process.^[^ ^9^ ^]^ Cells of both mating types, mt^+^ and mt^−^, start to produce sex‐inducing pheromones (SIP) as soon as they reach the sexual size threshold (SST) and become sexually active (**Figure** **3**). These SIPs are multifunctional and operate as synchronizing agents for sexual events by causing a cell cycle arrest in G1 phase in the opposite mating partner and thus increase mating efficiency.^[^ ^38^ ^]^ SIP^+^ of mt^+^ cells specifically triggers the production of the attraction pheromone cyclo(l‐pro‐l‐pro) (l‐diproline) in mt^−^.^[^ ^33^ ^]^ This first diatom pheromone has been identified and structurally elucidated based on a metabolomics‐guided approach and acts in low nanomolar concentrations to guide mt^+^ cells toward a compatible mt^−^ partner.^[^ ^39^ ^]^

In response to diproline, mt^+^ cells exhibit a chemokinetic acceleration and a chemotactic search behavior.^[^ ^40^ ^]^ A prerequisite is the SIP^−^‐induced formation of diproline receptors to ensure pheromone perception. On a molecular level, SIPs induce a change in gene expression in the opposite partner. Gene expression related to inhibition of cell cycle progression, chloroplast division, and cell wall formation was altered in both mating types, while genes involved in the diproline synthesis were only upregulated in the calling mt^−^. Two more genes encoding for transmembrane proteins were highly overexpressed in mt^+^ and qualify as genes encoding a putative diproline receptor.^[^ ^41^ ^]^

The planktonic pennate diatom *Pseudo‐nitzschia* *multistriata* pairs in the water column and shares similarities in its signaling chemistry with *S. robusta* (Figure 3, middle). Pheromones are secreted to synchronize the cell cycle in both mating types, and gametogenesis only happens when cells of the opposing mating types come in contact. However, no evidence of an attraction pheromone mediating pair finding was found. Substantial efforts of transcriptional and gene expression profiling led to the identification of mating type‐specific genes, including the sex‐determining gene *MRP3* in mt^+^ cells, and revealed gene expression changes during the whole process of sexual reproduction.^[^ ^42^, ^43^ ^]^

A similar, tightly orchestrated process is known in the freshwater araphid pennate diatom *Pseudostaurosira trainorii* (Figure 3, right). When mt^−^ cells get sexually active, they secrete a pheromone (ph‐1) that induces sexualisation of the partners. In response, these release two motile gametes and start to secrete a second pheromone that triggers the formation of eggs in mt^−^ cells. A third, very ephemeral pheromone is proposed to be secreted by sexualized mt^−^ cells to attract the gametes; however, none of these pheromones have been identified to date.^[^ ^29^ ^]^

## 4 Strategies for the Identification of Algal Sex Pheromones

The identification and elucidation of waterborn signaling cues like pheromones is often challenging due to their high chemical diversity and typically low abundance. The chemical nature ranges from highly polar to hydrophobic metabolites and includes volatiles as well as macromolecules,^[^ ^9^, ^19^ ^]^ thereby complicating the identification of unknowns. Already, finding an extraction and purification method for an unknown compound that is only produced in minute quantities is challenging. The subsequent isolation of the compound in sufficient amounts while completely removing inorganic salts for MS or NMR analysis often represents the major bottleneck.

For all approaches to the elucidation of sex pheromones, a robust bioassay to test the activity is a prerequisite. Bioassay development is often challenging, since the physicochemical nature of the pheromone, as well as the physiological response of the algae, have to be considered.^[^ ^44^ ^]^ Thus, for example, the hydrocarbon pheromones of brown algae (Figure 1) can be dissolved in a high‐density, chemically inert perfluorohexane, and the attraction of gametes to pheromone‐loaded beads can be observed under the microscope.^[^ ^10^ ^]^ The more polar diproline can be loaded on chromatographic resin beads and administered to cultures. Attraction of diatoms to the beads indicates that they contain the pheromones.^[^ ^45^ ^]^ For other algae, with unknown pheromones, specialized assays, like a capillary‐based delivery of attraction pheromone, have to be established and validated.^[^ ^27^ ^]^

**Figure 4:**
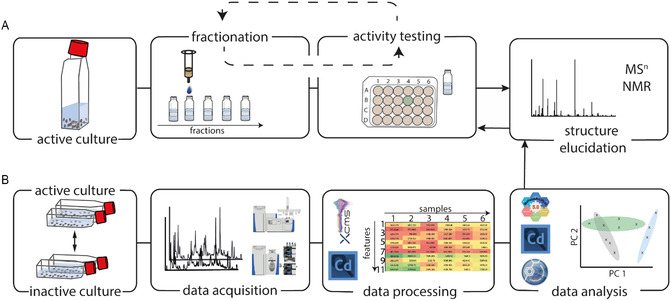
Identification of waterborn signaling cues. A) Bioassay‐guided fractionation includes an iterative process of fractionating and activity testing of an active extract. B) Depicts the metabolomics‐based approach comparing an active with a nonactive culture to identify pheromone candidates that are only detected in the active one. LC or GC‐MS metabolomics data and multivariate statistical analysis are required to identify the candidates that are then purified. The final step in both approaches is elucidation of the active compound by MS^n^ and/or NMR analysis and verification of its activity in bioassays.

The bioassay‐guided fractionation displays the classical method to identify active molecules among the huge number of compounds produced by an organism (**Figure** **4**A). The secreted active compound has to be extracted from the cell supernatant and purified mainly using chromatographic techniques. Separation by size, polarity, or charge of the compound is common.^[^ ^45^ ^]^ All fractions are tested for bioactivity. Further purification steps of the active fraction and bioactivity testing need to be conducted until the pure active compound is isolated. A subsequent structure elucidation based on standard analytical approaches like NMR supported by MS is required. When planning the scale of the experiments, one has to consider the loss of compound during the iterative process of purification and activity verification, as well as the long time periods needed to run a purification/activity testing step. Despite its limitations, this method has been applied successfully to identify pheromones to date.^[^ ^46^, ^47^ ^]^

The development of instruments that can provide analytical data of hundreds of compounds within an extract and of elaborate chemoinformatic methods dealing with the data has opened up new perspectives in pheromone research.^[^ ^48^, ^49^ ^]^ Especially high‐resolution MS analysis is useful for metabolomics‐based approaches to pheromone elucidation. Therefore, secreted metabolites (the exo‐metabolome) of the candidate organism in a sexually inactive and a sexually active state are compared to identify candidate metabolites that are upregulated in the active state.^[^ ^50^ ^]^ These can be isolated and tested without tedious repetition of purification/testing procedures. Data evaluation, however, is not trivial. First, the data are deconvoluted, which includes peak picking, peak alignment, and normalization of data. A list of features detected across the samples is generated. A feature is defined as a pair of mass to charge ratio (*m*/*z*) with a retention time. Advanced multivariate statistics are applied to investigate differences between samples. Open source software like XCMS^[^ ^51^ ^]^ performs preprocessing of the data and visualizes results of two‐group or multigroup comparisons in, e.g., cloud plots displaying fold changes of metabolites between different sample groups.^[^ ^52^ ^]^ Highly up‐ or down‐regulated features are candidates for testing in bioassays.

The comparative metabolomics approach was successfully applied in aquatic chemical ecology to investigate allelopathic interaction of marine dinoflagellates,^[^ ^53^ ^]^ bacteria–algal symbiosis^[^ ^54^ ^]^ or to characterize stress responses of the single‐celled green algae *C. reinhardtii* to nitrogen depletion.^[^ ^55^ ^]^ For a more detailed information on this approach, we refer to reviews that summarize the general application of metabolomics in chemical ecology and its use to discover new marine natural products.^[^ ^49^, ^56^, ^57^ ^]^

## 5 Identification and Structure Elucidation of Diatom Pheromones—The Case Study of *S. robusta*

Metabolomics studies that characterize developmental stages or pheromones involved in sexual reproduction of diatoms are mainly constraint to *Pseudo‐nitzschia multistriata* ^[^ ^58^ ^]^ and *S. robusta*.^[^ ^33^, ^38^ ^]^ *S. robusta* is the preferred model organism to study sexual reproduction in diatoms especially since the sexuality can be controlled under lab conditions.^[^ ^59^ ^]^ Genomic resources are available, and a suite of omics experiments was conducted to characterize sexual events.

**Figure 5:**
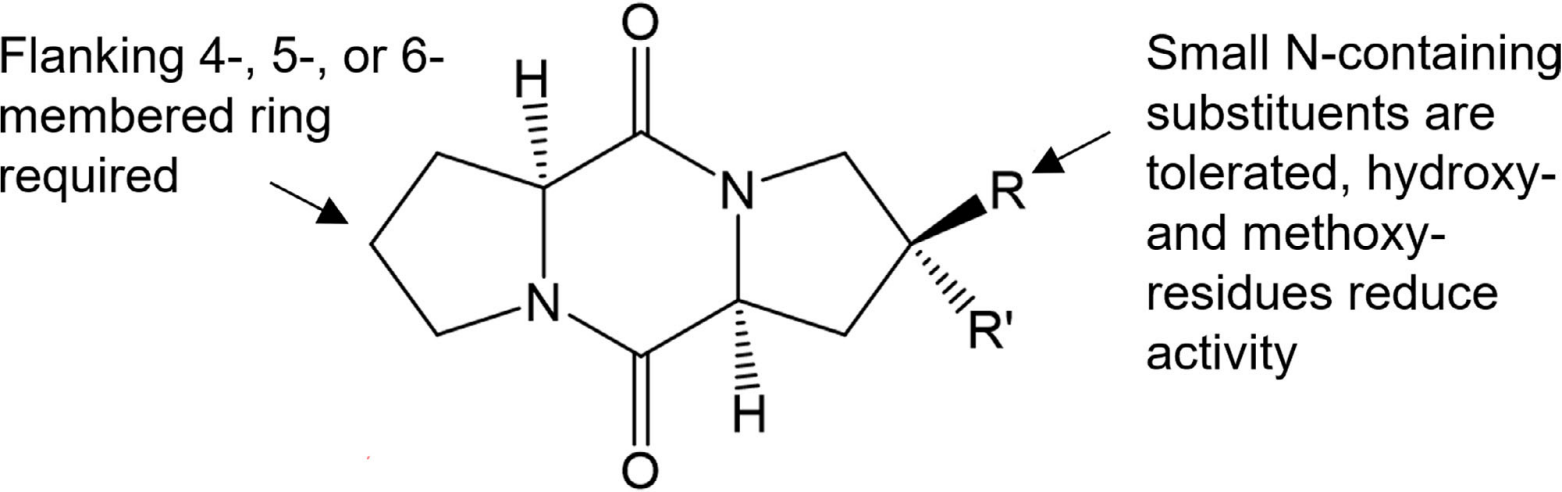
Results from structure–activity testing according to Moeys et al.^[^ ^66^ ^]^ and Lembke et al.^[^ ^39^ ^]^ While the diketopiperazine core is invariant, the five‐membered rings might be extended or reduced in ring size. Also, small substituents, but no other major modifications are tolerated.

For the structural elucidation of the first identified diatom pheromone, the attraction pheromone diproline (**Figure** **5**), a metabolomics approach was successful. MS‐based profiling of cell exudates of sexually mature mt^−^ cells in the presence and absence of culture supernatant of mt^+^ cells enabled the breakthrough. Mt^−^ only produces the attraction pheromone when the cells recognize the presence of a putative partner by sensing its SIP. Therefore, peaks were identified that only occur in the supernatant of cells treated with the SIP^+^‐containing medium from its calling partner in comparison to a control. Indeed, the statistically most significant up‐regulated peak proved to be the attraction pheromone that was identified after targeted fractionation and MS‐ and NMR‐based structure elucidation and supercritical fluid chromatography for enantiomer separation.^[^ ^45^, ^60^ ^]^ For activity testing, a simple but effective bioassay was developed, where the pheromone was loaded onto solid phase extraction cartridges. These were opened, and the resin beads loaded with the pheromone could be directly added to a culture of the searching partner. Counting the accumulated cells around the beads allowed to determine threshold concentrations. These pheromone‐loaded beads also allowed us to further analyze the movement patterns of the cells in the presence of the pheromones. The searching cells respond to the presence of the attraction pheromone with a combination of chemotaxis toward the bead and chemokinesis (i.e., speeding up of the cells) upon pheromone recognition.^[^ ^61^ ^]^ Interestingly, this attraction response is similar to the movement patterns when cells, starved of the nutrients silicate or phosphate, approach nutrient‐loaded beads.^[^ ^62^, ^63^ ^]^ The unicellular algae even show behavioral preference for nutrients when starved or for sex, when the cells reach a critically small size and need sexual reproduction for cell‐size restoration.^[^ ^64^ ^]^

Structure‐activity assays revealed that pheromone perception is a process with high specificity in *S. robusta*.^[^ ^39^ ^]^ Among nine diketopiperazines tested in attraction assays, only the pheromone diproline itself and a pipecolic acid‐derived diketopiperazine with two expanded aliphatic ring systems were active. Hydroxylation of the aliphatic rings abolished any bioactivity, and diketopiperazines derived from acyclic amino acids were not attractive as well. Remarkably, both enantiomers (*R*,*R*)‐ and (*S*,*S*)‐diproline were active, which was explained using structure modeling that revealed the possible orientation of the two enantiomers in a hypothetical enzymatic pocket. Very promising is the finding that an azidirene structural motif as a photoaffinity label can be introduced in the aliphatic part of the molecule without substantially losing activity, thereby opening up perspectives for the search of a first receptor protein in diatoms.^[^ ^65^ ^]^

**Figure 6:**
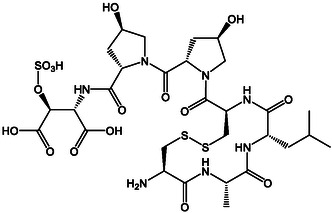
Structure of the SIP^+^ from the diatom *S. robusta.*

The identification of the first SIP in diatoms proved to be substantially more challenging. This stems from the fact that while the attraction pheromone diproline is released in comparably high nanomolar amounts upon demand SIPs are released continuously, but in very low concentrations, to indicate the presence of a sexually capable cell. Using comparative metabolomics, the first steps toward the elucidation of the SIP^+^ were undertaken, and a candidate peak could be identified that was uniquely present in sexually mature cells of mt^+^.^[^ ^66^ ^]^ The high molecular weight of this pheromone of 843 Da already indicated a higher structural complexity compared to the attraction pheromone. After tedious fractionation of hundreds of liters of culture medium, the isolated amount was still insufficient for NMR analysis. However, growing cultures in ^15^N‐labeled medium allowed to label all nitrogen atoms and enabled a detailed fragmentation analysis in liquid chromatography MS (LC‐MS/MS) studies. Growing the cells in ^13^C carbonate and ^15^N nitrate‐enriched medium allowed advanced NMR‐experiments including ^1^H, ^15^N‐ and ^1^H, ^13^C‐HMBC, even if less than 200 μg of SIP^+^ were available. The pheromone proved to be a cyclic heptapeptide with an unusual sulfated hydroxyaspartate and two hydroxylated proline amino acids (**Figure** **6**). We could estimate that a *S. robusta* cell produces ≈150–400 amol SIP^+^ within 5 days, summing up to low nanomolar concentrations in a dense culture.

Bioassays confirmed the activity of the pure compound, and a transcriptomic dataset revealed the impact of SIPs on gene expression of the opposite mating type, including cell cycle arrest and inhibition of chloroplast division,^[^ ^41^ ^]^ which complemented microscopic observations.

Within a short time after the report of the *S. robusta* SIP, a total synthesis of the pheromone, confirming the structural elucidation, was published.^[^ ^67^ ^]^ The solid‐phase peptide synthesis was challenging due to the structural motif of a sulfated hydroxyaspartate. The Malins‐lab introduced two synthetic approaches with an early and a late‐stage sulfation strategy, with the latter giving high yields and sufficient material to be used in future fieldwork or bioreactors.

## 6 Community Interactions

Sexual reproduction of *S. robusta* occurs in biofilms, which are complex multispecies communities. In this natural environment, pheromones represent targets for competition strategies by signal interference. Accordingly, sexual reproduction of *S. robusta* was most efficient in axenic cultures. In xenic cultures, bacteria from field‐collected biofilms slowly degraded the attraction pheromone and also modulated sexual reproduction by influencing the proportion of the different sexual states of the diatom. These effects were also observed with spent medium from the bacteria, indicating an intricate chemical cross‐species and even cross‐kingdom communication in biofilms.^[^ ^68^, ^69^ ^]^ A transcriptomics experiment demonstrated the positive and negative effects of bacteria on the sexual event by altering diatom gene expression.^[^ ^68^ ^]^ It will be, however, a subject of future studies to figure out if also interference strategies by other algae are shaping communities. These could involve targeted degradation of pheromones from competitors or the release of misleading signals or analogs that prevent mate finding. While we know from brown algal pheromone chemistry that there are a species‐specific pheromones involved, it is currently unclear if a similar chemical diversity is also found in diatoms. In fact, only *S. robusta* pheromones are elucidated today, and it will be a subject of future work to characterize the signaling chemistry of other species. It will also be challenging but rewarding to bring the results from laboratory assays to field work, which will reveal how communities are dependent and influenced by the chemical signals.

## 7 Conclusion and Outlook

We are only beginning to explore the pheromone chemistry of diatoms. With the work on the model species *S. robusta,* a starting point has been made that will support the investigation of further species. It will be interesting to see if and how the diversity of species is also reflected in their pheromone chemistry. With the pheromones in hand, we can now aim to obtain further insight into the fundamental questions of how a unicellular organism can perceive the pheromones and how this information is translated into physiological responses, such as directed movement patterns. In fact, to the best of our knowledge, till now no single pheromone receptor of any alga has been identified. It is also an open topic how the biosynthesis of the pheromones is achieved and how their production is regulated. Here, a joint approach using transcriptomics and metabolomics techniques might allow the breakthrough. But also, applications in large‐scale algal farming can be envisaged that might benefit from the targeted manipulation of cultures for the production of high‐value products.

## Conflict of Interest

The authors declare no conflict of interest.
